# Applicability of European legislation for the protection of data while using tracing applications

**DOI:** 10.1093/eurpub/ckac130.061

**Published:** 2022-10-25

**Authors:** P Mullerova

**Affiliations:** Faculty of Law, Lund University, Lund, Sweden

## Abstract

**Background:**

Since the beginning of the COVID pandemic, Member States have started using tracing applications. The European Commission immediately confirmed the possibility of collecting personal data without the obligation to obtain the citizens’ consent. Aware of the threat of data breach, the Commission has tried to remedy the situation and has given up the recommendation followed by a guide on how to use the tracing applications.

**Methods:**

In order to achieve the determination of the applicable European legislation, we used the doctrinal method in combination with the quantitative empirical research in the area of comparison of individual tracing applications.

**Results:**

The geolocal applications are regulated by GDPR. Based on Article 6 (1) GDPR, Commission confirmed the possibility to restrict the citizens’ privacy. The Commission drew the attention to the collection of personal data based on the exception of “public interest” without necessity of the citizens’ consent. In combination with Article 23, the Member states may restrict the lawful processing of data by legislative measure for protection of “public health”. The applications using Bluetooth exposure notification system (ENS) do not operate with personal data. Thus, these are in general out of scope GDPR and regulated by the Directive on privacy and electronic communications. Article 15 of the Directive allows processing of communication without citizens’ consent for the reasons listed in paragraph 1, which did not include “public health”, therefore the applications cannot be use without citizen's consent.

**Conclusions:**

The use of applications with geolocation allows Member states to process personal data without guarantees in order to protect public health. This approach is unacceptable in relation to the right to privacy. If a situation like the covid pandemic occurs again, exclusively applications with ENS should be used. The use of these applications, even in times of pandemic, is conditioned by the citizens’ consent.

**Key messages:**

- The project is expected to define applicable European legislation for using tracing applications and thus contributing to their safe use in the future with the least impact on citizens’ rights.
- The use of geolocal applications without legal proceeding of sensitive data contributed in 2020 to an increase of data breach up to 68%, thus it is necessary to adhere to the principles set by GDPR.

